# Rise in the Pathogenic Status of Coagulase-Negative Staphylococci Causing Bloodstream Infection

**DOI:** 10.7759/cureus.57250

**Published:** 2024-03-30

**Authors:** Girish Patil, Pragya Agarwala, Padma Das, Swati Pathak

**Affiliations:** 1 Microbiology, All India Institute of Medical Sciences, Raipur, Raipur, IND

**Keywords:** staphylococcus haemolyticus, vancomycin, paired blood cultures, bloodstream infection, coagulase-negative staphylococci

## Abstract

Background: Coagulase-negative staphylococci (CoNS) are one of the frequently isolated bacteria from blood cultures. Since they are part of the normal skin flora, they were previously considered contaminants. But now, they can be considered as established pathogens causing bloodstream infection (BSI). This study aims to estimate the prevalence of CoNS in BSI cases.

Methods: This study was conducted at the Microbiology Department, All India Institute of Medical Sciences (AIIMS), Raipur, India, for eight months (January 2022 to August 2022). Data were collected retrospectively from medical and laboratory records. Paired blood cultures from 5085 clinically suspected sepsis cases were subjected to aerobic culture for five days in the BacT ALERT 3D system. Pathogenicity was established after recovery of CoNS from paired blood cultures of symptomatic patients.

Results: CoNS were isolated from 2.35% of patients, the most common species being *Staphylococcus haemolyticus* (51.67%). About 90% of isolates were methicillin-resistant. All the isolates were susceptible to linezolid, teicoplanin, and vancomycin, except one isolate of *S. haemolyticus* which was intermediate to vancomycin. Minimum inhibitory concentration (MIC) 50 and MIC 90 for vancomycin were 1 ug/ml and 2 ug/ml, respectively.

Conclusion: Paired blood cultures are necessary to determine the pathogenicity of CoNS in BSI cases. A high prevalence of methicillin resistance, accompanied by high resistance rates to other non-beta lactam antibiotics, warrants the strict implementation of antimicrobial stewardship practices.

## Introduction

Bloodstream infections (BSI) are associated with significant morbidity and can be fatal if not treated promptly. Coagulase-negative staphylococci (CoNS) are one of the frequently isolated bacteria from blood cultures [1]. Since they are part of the normal skin flora, they were previously considered contaminants [2]. In recent years, they have been more frequently reported as a cause of bacteremia, especially in hospitalized patients [3]. It is essential to distinguish between a contaminant and a true pathogen to avoid unnecessary prescription of antimicrobials [4]. Determining the clinical significance of a single positive blood culture for CoNS is challenging since it is a part of the skin flora. Paired blood cultures are very helpful in establishing pathogenicity since the positive predictive value of paired blood cultures is 96-98% [5]. The increasing prevalence of CoNS bacteremia and the emergence of drug resistance among them, especially methicillin resistance, warrant their speciation and the determination of their antimicrobial resistance pattern [6].

This study was conducted to estimate the prevalence of various species of CoNS causing bacteremia and to study their antimicrobial susceptibility pattern.

## Materials and methods

Study design

This is an observational study where data is collected retrospectively from medical and laboratory records.

Study period

This study was conducted for eight months (January 2022 to August 2022) at the Microbiology Department, All India Institute of Medical Sciences (AIIMS), Raipur, India.

Processing of specimens

The aerobic culture was performed on paired blood specimens from 5085 patients with clinical suspicion of sepsis using the automated blood culture system BacT ALERT 3D (bioMérieux, Marcy-l'Étoile, France) for five days. Positively flagged bottles were sub-cultured on blood agar and MacConkey agar. Bacterial identification was performed using standard biochemical tests [7] and the Vitek 2 system (bioMérieux, Marcy-l'Étoile, France). Antimicrobial susceptibility testing was performed using the Kirby-Bauer disk diffusion method according to Clinical and Laboratory Standards Institute (CLSI) M100 [8] and the Vitek 2 system.

Inclusion criteria

All CoNS grown from paired blood cultures of clinically suspected sepsis cases were included in the study. CoNS were reported as pathogens only when isolated from paired blood specimens of symptomatic patients (body temperature >38℃ or <36℃ and hypotension). When just one blood specimen was available (particularly in neonates), along with symptoms, total leukocyte count (>12,000/µl or <2000/µl) [9,10] and procalcitonin [11] were evaluated before establishing pathogenicity.

Exclusion criteria

The following blood culture isolates were excluded from the study before analysis: (a) CoNS isolated from a single blood culture, (b) all bacteria other than CoNS, and (c) yeasts and yeast-like fungi.

Statistical analysis

All data collected were entered and analyzed using Microsoft Excel. Categorical variables were expressed as percentages. P-value was calculated using the chi-squared test using the Epi Info software; values <0.05 were considered statistically significant.

Ethical approval was not required, since the blood specimens were collected as a part of patient care and the data was collected retrospectively from medical records.

## Results

CoNS were isolated from about 2.35% (n=120) of 5085 patients. The most common age group affected was 41-60 years (33.34%), followed by >60 years (18.34%), with male preponderance. Around 7.5% of isolates were from infants and 11.67% from newborns (Table 1).

**Table 1:** Age and sex distribution of patients with bacteremia due to CoNS CoNS: coagulase-negative staphylococci

Age group	Male n (%)	Female n (%)	Total
Neonate	8 (6.67)	6 (5)	14 (11.67%)
Infant	6 (5)	3 (2.5)	9 (7.5%)
1-20	10 (8.34)	4 (3.34)	14 (11.67%)
21-40	9 (7.5)	12 (10)	21 (17.5%)
41-60	24 (20)	16 (13)	40 (33.34%)
>60	11 (9.16)	11 (9.16)	22 (18.34%)
Total	68 (56.67)	51 (42.5)	120

The proportion of CoNS recovered from patients admitted to wards (64.16%) was higher than that in intensive care units (32.5%) and outpatient clinics (3.34%). The highest number of CoNS were recovered from patients from General Medicine wards (20.83%, n=25), followed by the Trauma and Emergency Department (17.5%, n=21) and the Neonatal Intensive Care Unit (11.67%, n=14) (Table 2).

**Table 2:** Distribution of CoNS among different departments and locations CoNS: coagulase-negative staphylococci; ICU: intensive care unit; COVID-19: coronavirus disease 2019

Ward	Number of CoNS isolates	Percentage (%)
General Medicine	25	20.83
Trauma and Emergency	21	17.5
Neonatal ICU	14	11.67
Paediatric ICU	9	7.57
Nephrology	9	7.57
Paediatrics	6	5.00
Dermatology	6	5.00
Critical Care Unit	5	4.16
COVID-19 ICU	5	4.16
Medical ICU	4	3.34
General Surgery	4	3.34
Outpatient Department	4	3.34
Orthopaedics	3	2.5
Neurology	1	0.83
Urology	1	0.83
Cardiovascular and Thoracic Surgery	1	0.83
Pulmonary Medicine	1	0.83
Otolaryngology	1	0.83
Total	120	

Five isolates of CoNS were obtained from a single blood sample of neonates which were ascertained pathogens based on clinical and laboratory parameters mentioned above.

**Figure 1:**
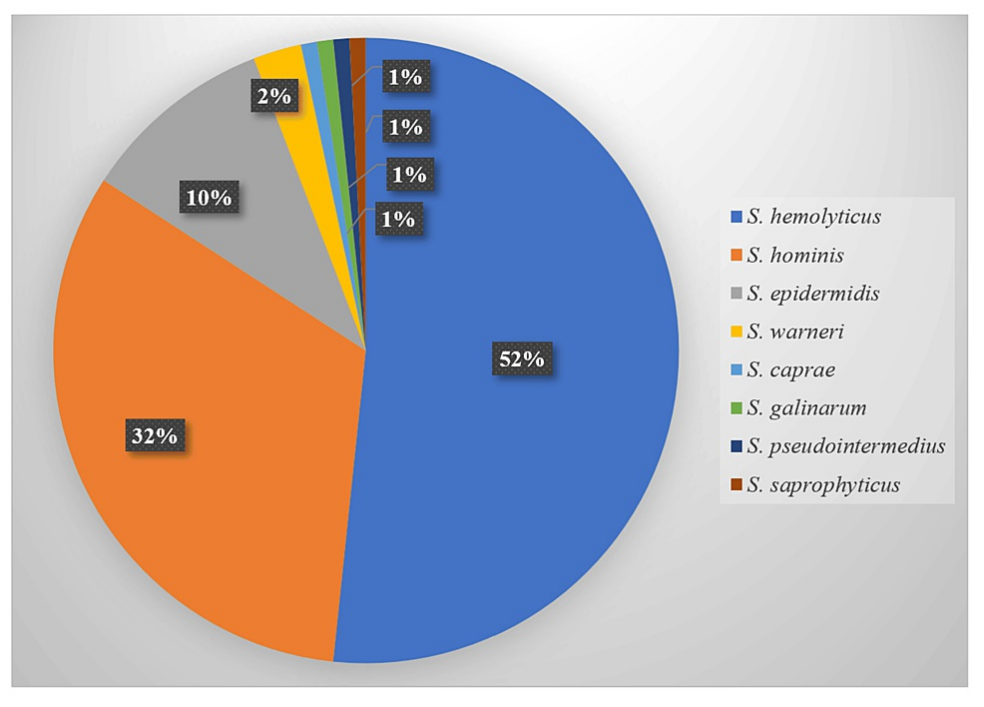
Species distribution of isolated CoNS CoNS: coagulase-negative staphylococci

*Staphylococcus haemolyticus* (n=62, 51.67%) was the most commonly isolated species, followed by *S. hominis *(n=39, 32.5%) and *S. epidermidis* (n=12, 10%) (Figure 1).

**Figure 2:**
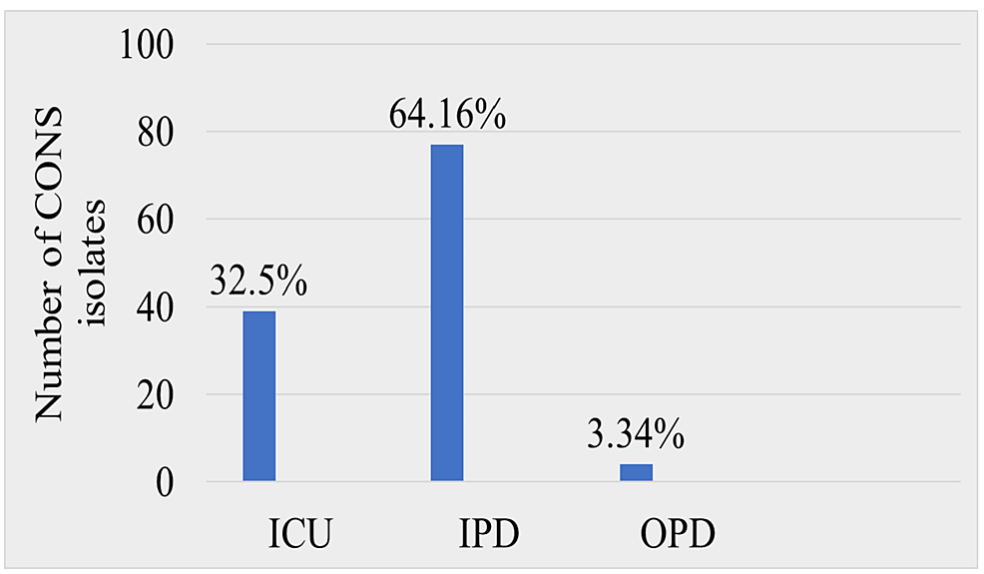
Distribution of CoNS among ICU, IPD, and OPD CoNS: coagulase-negative staphylococci; ICU: intensive care unit; IPD: in-patient department; OPD: out-patient department

Overall, the percentage of CoNS isolated from in-patients (64.16%) was higher as compared to patients admitted to intensive care units (32.5%) and out-patients (3.34%) (Figure 2). 

Almost 90% of the isolates showed methicillin resistance (MRCoNS). Higher resistance to non-beta lactam antimicrobials was also seen in these MRCoNS compared to methicillin-resistant CoNS (MSCoNS). Erythromycin showed the highest resistance (91.57%), followed by levofloxacin (82.4%), ciprofloxacin (73.8%), and co-trimoxazole (74.7%). Doxycycline showed the lowest resistance (20.56%). All the isolates were 100% susceptible to vancomycin, teicoplanin, and linezolid, except one *S. haemolyticus* isolate, which was intermediate to vancomycin (minimum inhibitory concentration (MIC): 8 ug/ml) (Table 3).

**Table 3:** Resistance profile of MRCoNS and MSCoNS to non-beta lactam antimicrobials *Inducible clindamycin resistance 17 (15.88%) All the isolates were susceptible to linezolid, teicoplanin, and vancomycin except one isolate which was intermediate to vancomycin (MIC: 8 ug/ml) P-value <0.05 is considered statistically significant MRCoNS: methicillin-resistant coagulase-negative staphylococci; MSCoNS: methicillin-sensitive coagulase-negative staphylococci; MIC: minimum inhibitory concentration

Antimicrobial	MRCoNS (107) n (%)	MSCoNS (13) n (%)	P-value (<0.05)
Erythromycin	98 (91.57)	09 (69.23)	0.014
Clindamycin*	62 (57.94)	00	0.0007
Co-trimoxazole	80 (74.76)	02 (15.38)	0.00014
Doxycycline	22 (20.56)	01 (7.69)	0.2656
Ciprofloxacin	79 (73.83)	02 (15.38)	0.00002
Levofloxacin	88 (82.24)	03 (23.07)	0.00001
Gentamicin	40 (37.38)	00	0.0069

MIC 50 and 90 values for oxacillin, vancomycin, linezolid, and teicoplanin were also calculated, as depicted in Table 4.

**Table 4:** MIC of oxacillin, vancomycin, linezolid, and teicoplanin for isolated CoNS MIC: minimum inhibitory concentration; CoNS: coagulase-negative staphylococci

Antimicrobials	MIC range (ug/ml)	MIC 50	MIC 90	% resistance
Oxacillin	0.25-≥4	4	≥4	89.16
Vancomycin	0.5-8	1	2	0
Linezolid	1-4	2	4	0
Teicoplanin	0.5-8	2	4	0

## Discussion

CoNS are increasingly being reported from BSI cases. Understanding the difference between contamination and infection will help to reduce the inappropriate use of antimicrobials. The prevalence of CoNS bacteremia in the present study is 2.35%, which is similar to the analysis conducted by Singh et al. (2.8%) [9] but lower than the prevalence observed by Singh et al. (9.78%) [12]. A greater prevalence of CoNS bacteremia (25-57%) [13] has been observed in Western countries. The increased use of invasive medical devices may account for this difference.

In this study, *S. haemolyticus *was the most frequently encountered pathogen, similar to results found by Singh et al. [9] and Jain et al. [14]. However, Usha et al. [15] and Singh et al. [12] found that *S. epidermidis* was the most common species, followed by *S. haemolyticus*. The skin flora of the medical personnel and patients might have influenced these results.

Patients admitted in wards were more affected than in ICUs. Similar results were obtained by Singh et al. [12]. This may be because the number of patients admitted in wards is higher and infection control practices are strictly followed more in ICUs as compared to wards.

About 90% of the isolates in this investigation were resistant to methicillin, and many also showed elevated resistance to other non-beta lactam antibiotics. Singh et al. [12] got similar findings. Increasing MRCoNS incidence is concerning because it reduces available therapeutic options. Also, the resistance genes could spread to other staphylococcal strains in the hospital environment or on the patient's skin.

The isolates displayed 100% sensitivity to vancomycin, linezolid, and teicoplanin, which has been reported in a study from Lucknow, too [12]. In a Brazilian study, 2.7% of isolates were found to have decreased susceptibility to vancomycin [16]. In contrast, Yamada et al. from Japan found 20% resistance to teicoplanin and 100% susceptibility to linezolid and vancomycin [17], and in an Italian study, 5.4% of CoNS isolates showed decreased susceptibility to vancomycin [18].

In the present study, MIC 50 and MIC 90 for vancomycin were 1 ug/ml and 2 ug/ml, respectively. Our findings are in keeping with previous reports by Pereira et al. [16] and Yamada et al. [17]; both got values of 1.5 ug/ml and 2 ug/ml, respectively. For linezolid, MIC 50 and 90 values were 2 ug/ml and 4 ug/ml, respectively. However, this is higher than the values reported by Pereira et al. [16] (0.25 ug/ml and 0.5 ug/ml) and Yamada et al. [17] (0.75 ug/ml and 1 ug/ml), respectively. For teicoplanin, MIC 50 and 90 values were 2 ug/ml and 4 ug/ml, respectively. But higher values were reported by Yamada et al. [17] (8 ug/ml and 16 ug/ml) for teicoplanin. 

Strengths and limitations

We have calculated MIC 50 and 90 values for vancomycin, linezolid, and teicoplanin. 

Our study period was from January to August 2022. We have analyzed data for only eight months. We were not able to analyze seasonal variations.

Only the phenotypic method was used for the detection of methicillin resistance, as per CLSI guidelines 2022. We have not detected the mecA gene for methicillin resistance.

## Conclusions

It is important to obtain paired blood cultures to determine the pathogenicity of CoNS in symptomatic patients. When only a single blood culture is available, other relevant parameters like total leukocyte count and procalcitonin help to establish the pathogenicity.

A high prevalence of methicillin resistance, accompanied by high resistance rates to other non-beta lactam antibiotics, warrants the strict implementation of infection control and antimicrobial stewardship practices. Antibiotics such as vancomycin, linezolid, and teicoplanin should be used judiciously to prevent the development of drug resistance.
